# Supplementary material for: Chitosan Enhances the Anti-Biofilm Activity of Biodentine against an Interkingdom Biofilm Model
Source: Antibiotics (Basel). 2021 Oct 29;10(11):1317. doi: 10.3390/antibiotics10111317 (PMC8614659; doi:10.3390/antibiotics10111317)
Supplement: Supplementary file 1 [file antibiotics-10-01317-s001.zip › antibiotics-1427125-supplementary.pdf]

# Supplementary Materials

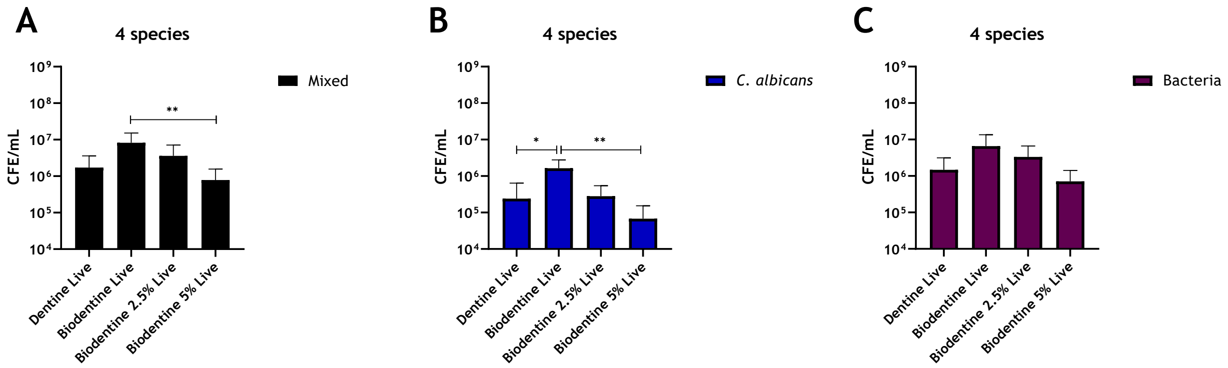


**Figure S1.** Compositional analysis of regrown biofilms on Biodentine material discs. Live/dead qPCR was performed on biofilms following incorporation of 2.5 wt% and 5 wt% chitosan into Biodentine material. (A) Live CFE/mL of 4-mixed biofilms (bacteria & *Candida*). (B) Live CFE/mL of *C. albicans* in mixed species biofilms. (C) Live CFE/mL of bacteria in mixed species biofilms. Bovine dentine and unaltered Biodentine discs were used as controls. Data were analysed by Kruskal-Wallis with Dunn's tests. * Indicates statistically significant differences (* $p < 0.05$, ** $p < 0.01$).
